# CGRP monoclonal antibody for preventive treatment of chronic migraine: An update of meta‐analysis

**DOI:** 10.1002/brb3.1215

**Published:** 2019-01-18

**Authors:** Lin Han, Yao Liu, Hai Xiong, Peiwei Hong

**Affiliations:** ^1^ Department of Geriatrics West China Fourth Hospital, Sichuan University Chengdu China; ^2^ Xindu Hospital of Traditional Chinese Medicine Chengdu China; ^3^ Tibet University Medical College Lasha China

**Keywords:** calcitonin gene‐related peptide, CGRP, chronic migraine, meta‐analysis

## Abstract

**Background:**

CGRP monoclonal antibody (mAb) is a promising preventive treatment for episodic migraine and has been approved by US FDA recently. But the treatments for chronic migraine are rare. Therefore, we performed meta‐analysis to assess the efficacy and safety of CGRP mAbs in preventing chronic migraine.

**Methods:**

Database including Cochrane Library and PubMed were systematically searched for randomized controlled trials (RCTs) which are about CGRP mAb in preventing treatment of chronic migraine. Evaluating the bias and quality of RCTs was carried out according to the Cochrane collaboration's tool for assessing risk of bias. The data analysis was carried out by reviewer manager 5.2.

**Results:**

Totally, 6 articles enrolled in the present meta‐analysis, including 4 independent clinical trials and 3,166 patients. After pooled analysis, it indicated that CGRP mAb improved 50% responder rate (OR = 2.42, 95% CI = [2.04, 2.87], *I* ^2^ = 0%, *p* < 0.00001) and 75% responder rate (OR = 1.95, 95% CI = [1.30, 2.91], *I* ^2^ = 0%, *p* = 0.001), as compared with placebo. And there was no difference in incidence of adverse events between CGRP mAb group and placebo group except incidence of injection site discomfort.

**Conclusions:**

CGRP mAb is an effective and safety preventive treatment for chronic migraine.

## 1 INTRODUCTION

Chronic migraine is a disabling primary headache disorder, defined as headache occurring no less than 15 days per month for more than three months and has the features of migraine headache for no less than eight days per month according to the International Classification of Headache Disorders, 3rd edition (ICHD‐3) (Headache Classification Committee of the International Headache Society, 2013). Approximately 2% of the population occurs chronic migraine, which leads to lower health‐related quality of life and functional impairment as compared with episodic migraine (Bigal et al., 2015; Silberstein et al., 2017; Tepper et al., 2017). Patients with chronic migraine are more likely suffering from be divorced, be unemployed psychological comorbidity, and high risk with acute medication overuse for headache treatment (Bigal et al., 2015; Silberstein et al., 2017; Tepper et al., 2017). The expert opinion suggests that patients with chronic migraine should receive abortive and preventive treatments (Giacomozzi et al., 2013; Irimia, Carmona‐Abellan, & Martinez‐Vila, 2012; Lionetto et al., 2012; Pringsheim et al., 2012; Puledda, Messina, & Goadsby, 2017). But there are few treatments for preventing chronic migraine. And the onabotulinum toxin A and topiramate are the class I drugs with level A evidence (Giacomozzi et al., 2013). Sodium valproate, gabapentin, pregabalin, amitriptyline, tizanidine, and methysergide are the alternative preventive treatments for chronic migraine with lower evidence levels (Giacomozzi et al., 2013). Although about 40% migraineurs would benefit from preventive treatments, only a minority receive it because of treatment failure, resulting from lack of efficacy and adverse events (Reuter, 2018).

Calcitonin gene‐related peptide (CGRP) is a promising target to treat migraine, which has a crucial role in migraine pathophysiology. And CGRP antagonisms and CGRP monoclonal antibody (mAb) are the drugs to inhibit the CGRP pathway resulting in the migraine treatment (Deen et al., 2017; Deneris, Rosati Allen, Hart Hayes, & Latendresse, 2017; Messlinger, 2018; Negro, Lionetto, Simmaco, & Martelletti, 2012; Schuster & Rapoport, 2017; Yuan, Lauritsen, & Kaiser, 2017). Some of CGRP antagonisms related clinical trials are discontinued because of the liver injury after repeated administrations (Hong & Liu, 2017; Hong, Wu, & Liu, 2017). CGRP mAb is a promising treatment for migraine prevention, which include eptinezumab (ALD403), erenumab (AMG 334), galcanezumab (LY2951742), and fremanezumab (TEV‐48125) (Yuan et al., 2017). And erenumab has been approved by U.S. Food and Drug Administration in preventing episodic migraine. Here we evaluate the efficacy and safety of CGRP mAbs in preventing chronic migraine with meta‐analysis.

## 2 METHODS

### 2.1 Data selection

Database including Cochrane Library and PubMed were queried using the following terms: migraine, calcitonin gene‐related peptide, and CGRP. The cutoff date was 4 July 2018, and we would pay attention to the advance of this area and revise our data before the manuscript has been published.

The literatures of randomized controlled trials (RCTs) in English and matching the following criteria were enrolled: (a) Patients diagnosed for chronic migraine according to the ICHD‐3 and (b) intervention is CGRP mAb.

### 2.2 Data extraction and analysis

The literatures screening and quality and bias of RCTs assessing are described in our previous articles (Hong & Liu, 2017; Hong et al., 2017). The primary efficacy outcomes included 50%, 75%, or 100% responder rate, defined as a at least 50%, 75%, or 100% reduction in monthly headache/migraine‐days from baseline to weeks 9–12. The secondary efficacy outcomes included change of migraine‐days from baseline to weeks 9–12, change of days using acute drugs from baseline to weeks 9–12, and change of headache‐hours from baseline to weeks 9–12. The safety outcomes were incidence of adverse events after drug administrated.

### 2.3 Data analysis

Data analysis was described in our previous articles (Hong & Liu, 2017; Hong et al., 2017). In briefly, Review manager 5.3 (Cochrane Collaboration) is utilized for data analysis and the threshold P value less than 0.05 was set as the significant level.

## 3 RESULTS

**Figure 1:**
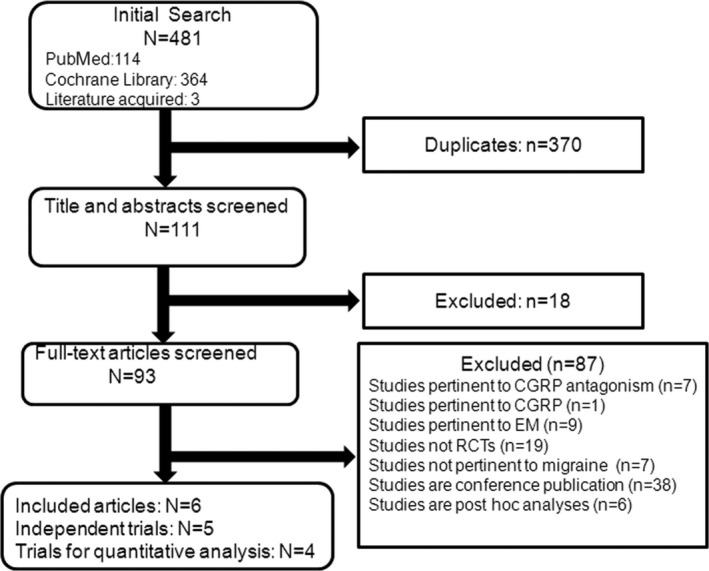
Flowchart

According to the retrieval strategy, we found 481 literatures; 6 articles were left after removal of the repetition and unmatched articles. The screening process was presented in Figure 1. The 6 articles included 2 phase 2b complete RCTs (Bigal et al., 2015; Tepper et al., 2017), 2 phase 3 complete RCTs (Detke et al., 2018; Silberstein et al., 2017), and 1 phase 3 ongoing RCT (Smith et al., 2017). So we analyzed the 4 complete trials with 3,166 chronic migraineurs. Out of 3,166 patients, 1,862 patients received the CGRP mAb, and the rest 1,304 patients received placebo. Erenumab was used in 1 trial with 70 mg or 140 mg subcutaneous every 4 weeks for 12 weeks (Tepper et al., 2017). Fremanezumab was used in 2 trials with different usage for 12 weeks (Bigal et al., 2015; Silberstein et al., 2017). And the galcanezumab was used in 1 trial with 120 mg or 240 mg subcutaneous every 4 weeks for 12 weeks (Detke et al., 2018). The detail information is shown in Table 1. Among above mentioned, erenumab is a human monoclonal antibody against CGRP receptor, fremanezumab, and galcanezumab are monoclonal antibody against CGRP ligand.

**Table 1:** The characteristic of enrolled trials

Trial ID	Interventions drug	Binding sites	Experimental	Administration route
Tepper 2017	Erenumab (AMG334)	CGRP receptor	70/140 mg	subcutaneous every 4 weeks for 12 weeks
Bigal 2015	Fremanezumab (TEV‐48125)	CGRP	225, 675 mg	subcutaneous 675 mg in the first treatment cycle and 225 mg in the second and third treatment cycles^a^
			900 mg	subcutaneous 900 mg in three treatment cycles
Silberstein 2017	Fremanezumab (TEV‐48125)	CGRP	225 mg	Subcutaneous 225 mg monthly for 12 weeks
			675 mg	subcutaneous 675 mg quarterly for 12 weeks
PROMISE 2 trial	Eptinezumab (ALD403)	CGRP	300/100/30/10 mg	A single intravenous dose for 12 weeks
REGAIN study	Galcanezumab (LY2951742)	CGRP	120 mg	subcutaneous 120 mg (with 240 mg loading dose)monthly for 12 weeks
			240 mg	subcutaneous 240 mg monthly for 12 weeks

a One treatment cycle is 28 days.

In the 5 independent trials, all of the terms of risk bias are low risk except the PROMISE 2 trial according to tool for assessing risk of bias in the Cochrane handbook. Therefore, the enrolled trials were of high quality. The detail information was shown in Table 2.

**Table 2:** Risk of bias of enrolled trials

Study ID	Tepper 2017	Bigal 2015	Silberstein 2017	PROMISE 2 trial	REGAIN study
Random sequence generation	Low risk	Low risk	Low risk	Unclear risk	Low risk
Allocation concealment	Low risk	Low risk	Low risk	Unclear risk	Low risk
Blinding of participants and personnel	Low risk	Low risk	Low risk	Unclear risk	Low risk
Blinding of outcome assessment	Low risk	Low risk	Low risk	Unclear risk	Low risk
Incomplete outcome data	Low risk	Low risk	Low risk	Unclear risk	Low risk
Selective reporting	Low risk	Low risk	Low risk	Unclear risk	Low risk
Other bias	Low risk	Low risk	Low risk	Unclear risk	Low risk

**Figure 2:**
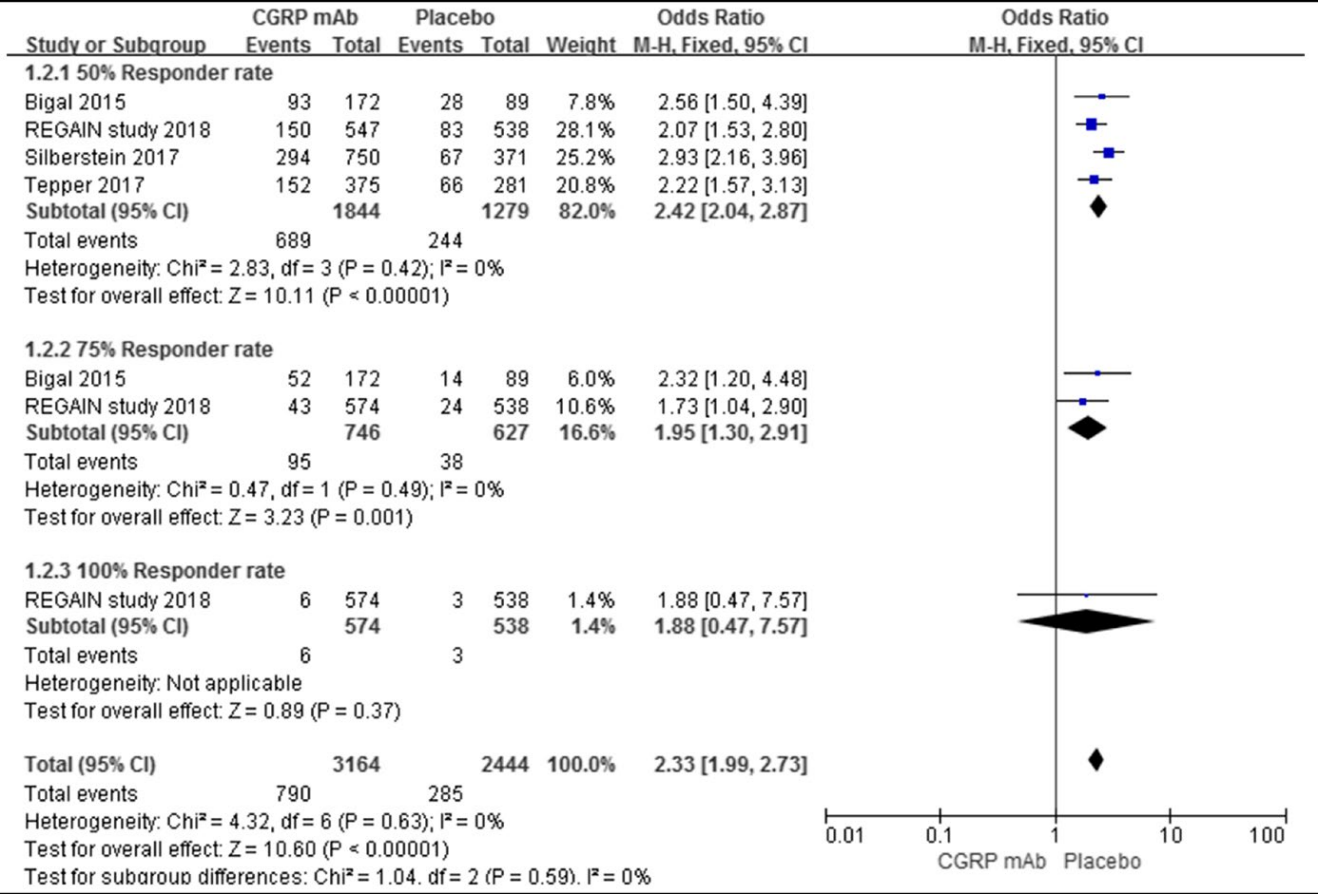
Responder rate

Regard with the primary efficacy outcomes, there were 4 trials had reported the 50% responder rate. And 37.4% (689/1844) achieved the 50% responder rate in CGRP mAb group, which was superior than 19.1% (244/1,279) in placebo group (OR = 2.42, 95% CI = [2.04, 2.87], *I* ^2^ = 0%, *p* < 0.00001). Only 2 trials had reported the 75% responder rate. And 12.7% (95/746) achieved the 75% responder rate in CGRP mAb group, which was superior than 6.1% (38/627) in placebo group (OR = 1.95, 95% CI = [1.30, 2.91], *I* ^2^ = 0%, *p* = 0.001). There was only 1 trial reported the 100% responder rate, but there was no difference between CGRP mAb group and placebo group(OR = 1.88, 95% CI = [1.99, 2.73], *p* = 0.37). The results were shown in Figure 2.

**Figure 3:**
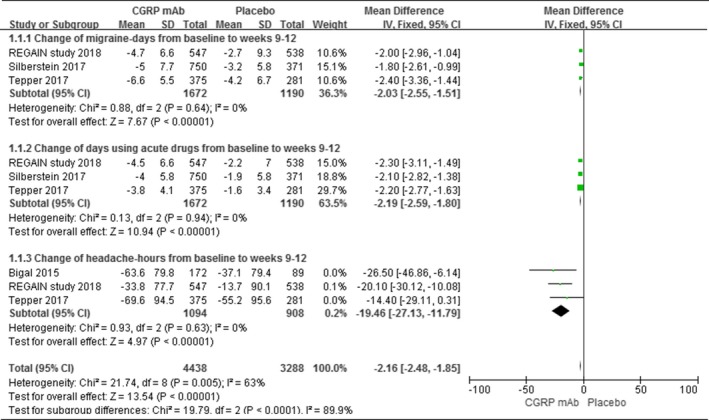
Secondary efficacy outcomes

Regarding the secondary efficacy outcomes, three trials had reported the change of migraine‐days from baseline to weeks 9–12. We found that the erenumab, fremanezumab, and galcanezumab have significant difference in this clinical index, as compared with placebo. After pool estimated, the CGRP mAb was larger than placebo in change of migraine‐days from baseline to weeks 9–12 (WMD = −2.03, 95% CI = [−2.55, −1.51], *I* ^2^ = 0%, *p* < 0.00001). And the same results were acquired in change of days using acute drugs from baseline to weeks 9–12 (WMD = −2.19, 95% CI = [−2.59, −1.80], *I* ^2^ = 0%, *p* < 0.00001) and change of headache‐hours from baseline to weeks 9–12 (WMD = −19.46, 95% CI = [−27.13, −11.79], *I* ^2^ = 0%, *p* < 0.00001).The results were shown in Figure 3.

Regarding the safety of CGRP mAb, we found that the incidence of injection discomfort was 676/1862 in CGRP mAb group, which is greater than 290/1,304 in placebo group (OR = 2.11, 95% CI = [1.37, 3.26], *I* ^2^ = 59%, *p* = 0.0007). Meanwhile, we found that the incidence of liver injury was higher in CGRP mAb group without significant difference, as compared with placebo (OR = 2.09, 95% CI = [0.65, 6.75], *I* ^2^ = 0%, *p* = 0.21). And we assessed discontinuation due to the adverse events, nausea, and the infection/inflammation‐related adverse events and found that there are no difference in the incidence of discontinuation, nausea, upper respiratory tract infection, sinusitis, and urinary tract infection between CGRP mAbs and placebo. The results were summarized in Table 3.

**Table 3:** The summary of adverse events

Adverse events	CGRP mAb (*n*/*N*)	Placebo (*n*/*N*)	*I* ^2^	Odds ratio [95% CI]	*p* (M‐H)
Injection discomfort	676/1,862	290/1,304	59%	2.11 [1.37, 3.26]	0.0007
Discontinuation	26/1,862	17/1,304	0%	0.96 [0.51, 1.79]	0.89
Liver injury	14/1,484	5/1,022	0%	1.53[0.54, 4.33]	0.42
Nausea	20/1,133	18/657	42%	0.66 [0.35, 1.26]	0.21
Upper respiratory tract infection	63/1,688	32/1,215	0%	1.34 [0.87, 2.08]	0.18
Nasopharyngitis	71/1,862	66/1,304	39%	0.75 [0.53, 1.06]	0.10
Sinusitis	30/1,484	16/1,022	47%	1.23 [0.67, 2.25]	0.51
Urinary tract infection	16/1,107	9/929	0%	1.46 [0.64, 3.29]	0.37

## 4 DISCUSSION

The present meta‐analysis assesses efficacy and safety of CGRP mAb in preventing chronic migraine by meta analyze 4 RCTs with high quality. The meta‐analysis demonstrates that CGRP mAb led to improvement in 50% responder rate, 75% responder rate, migraine‐days, days using acute drugs and headache‐hours after CGRP mAb administrated and well tolerated, as compared with placebo. These results are in line with previous published RCTs results.

The CGRP mAbs included in present study were administrated subcutaneous. And the injection site discomfort was the most common adverse event. And this discomfort included pain, pruritus, erythema, induration, edema, and bruising (Bigal et al., 2015; Detke et al., 2018; Silberstein et al., 2017; Tepper et al., 2017). In REGAIN study, one patient in CGRP mAb group discontinued because of the injection site pain (Detke et al., 2018). In the present meta‐analysis, we found that patients in CGRP mAb group experienced more injection site discomfort than placebo group, but the incidence of discontinuation due to adverse event were similar between these two groups. These results demonstrate that CGRP mAb is a well‐tolerated drug.

Liver function impairment was the most serious problems of CGRP receptor antagonisms in preventing migraine (Hong & Liu, 2017; Hong et al., 2017). But in the CGRP mAbs for episodic migraine clinical trials, there was no drug‐associated hepatotoxicity had been reported (Hong et al., 2017). In the present analysis, we found that 14/1,484 patients in CGRP mAb group and 5/1,022 patients in placebo group suffer from liver injury, but there is no significant difference. Bigal et al. (2015) reported that four patients had transient liver enzyme increasement with nontreatment related. But Silberstein et al. (2017) found that 10 patients suffered from possible trial‐agent–induced liver injury in CGRP mAbs group and three patients in placebo group. And eight patients in CGRP mAb group and three patients in placebo group had liver enzyme level <3–5 times the upper limits of normal range and had used acetaminophen or nonsteroidal anti‐inflammatory drugs frequently or antidepressants daily (Silberstein et al., 2017). Two patients in CGRP mAb group suffered from alanine transaminase or aspartate aminotransferase level more than five times the normal range's upper limit, one patient's aspartate aminotransferase level was normalized without intervention, and the other was normalized after the ethanol‐containing drug was discontinued (Silberstein et al., 2017). The REGAIN study had reported 2 patients suffering from abnormal hepatic enzyme (1 in placebo group and 1 in galcanezumab 240 mg group), and the patient in CGRP mAb discontinued (Detke et al., 2018). These results demonstrated that CGRP mAb might not lead to liver injury, the real‐world data would be monitored in future.

Another Important aspect of CGRP mAb is the incidence of infection. Silberstein et.al. found that 34/755 patients in mAbs suffered from upper respiratory tract infection and nasopharyngitis, 14/755 patients experienced sinusitis, but there is no significant difference between CGRP mAbs and placebo (Silberstein et al., 2017). Tepper et al. (2017) found that 11/378 patients suffered from upper respiratory tract infection, as compared to 4/282 patients in placebo. And there is no difference in incidence of urinary tract infection. Even, the incidence of nasopharyngitis was lower in CGRP mAbs, as compared to placebo (Tepper et al., 2017). Bigal et al. (2015) found that there are no differences in incidence of nasopharyngitis, sinusitis, and urinary tract infection between these two groups. The REGAIN study had reported that there were no difference between CGRP mAb group and placebo group in the incidence of upper respiratory tract infection, nasopharyngitis, and urinary tract infection. But the incidence of sinusitis in galcanezumab 240 mg group was greater than placebo group (Detke et al., 2018). In the present meta‐analysis, we found that there are no differences in the incidence of upper respiratory tract infection, sinusitis, and urinary tract infection between these two groups. Even, the incidence of nasopharyngitis is lower in CGRP mAb, as compared with placebo. These results demonstrated that CGRP mAb is safety and well tolerated for chronic migraine which admitted for further study and real‐world application.

The limitation of the present meta‐analysis was the lack of the best dose or regimen of CGRP mAbs for preventive treatment of chronic migraine because of the small sample size. Further trials with larger sample and real‐world data should be estimated in the future.

In conclusion, CGRP mAb is an effective and safety preventive treatment for chronic migraine.

## CONFLICT OF INTEREST

The author declares that he has no conflict of interests.

## AUTHORS’ CONTRIBUTIONS

All author read and approved the final manuscript.
